# Head Lice at School: Traditional Medicine and Community Engagement

**DOI:** 10.1089/heq.2020.0065

**Published:** 2021-05-13

**Authors:** Renata Campos Nogueira, Fabiana Regina Nonato, Maria Cristina Duchene Veauvy, Anne-Laure Cavin, Marwah Al-Anbaki, Bertrand Graz

**Affiliations:** ^1^ Antenna Foundation, Geneva, Switzerland.; ^2^ Byos Solues Tecnolgicas, Campinas, Brazil.

**Keywords:** head lice, whole-community approach, traditional knowledge, school program

## Abstract

**Purpose:** The prevalence of head lice in poor rural communities and urban slums is estimated to be between 28% and 43% in Brazil, respectively. Children are among the most affected, often in clusters within schools. We launched a program intending to tackle the social stigma associated with head lice using scientific information and a local traditional remedy as a way to lower the prevalence of head lice in a low-resource community.

**Methods:** A program involving the entire school community and the teachers addressed how to treat head lice and avoid new infestations. An affordable solution widely used in traditional Brazilian medicine was provided for the ones infested. Evaluation of the outcome was based on direct observation and was designed as a satisfaction survey. The study complied with the criteria for Standards for Reporting Qualitative Research (SRQR).

**Results:** Two hundred and eighty participants, including parents and siblings of the school children, took part in the program. Among them, 24% (*N*=67) had head lice, with girls representing 85% of cases; 74.7% of participants infested with head lice were between 4 and 10 years old; 55.2% (*N*=37) of participants infested showed no signs of nits or adult lice after the program.

**Conclusions:** This experience suggests that the use of playful activities associated with a well-known and accessible local product to treat head lice in low-income families gathered a high degree of community adherence and may be an important tool in overcoming health inequalities.

## Introduction

Pediculosis caused by head lice (*Pediculus humanus var capitis*) infestation is widespread worldwide.^1^ Transmission mainly occurs through physical head-to-head contact and is highly influenced by the population density of potential host.^2^ Therefore, households composed of a large number of children and living in densely populated municipalities present a higher prevalence of infestation. Schools, refugee camps, jails, orphanages, and any type of overcrowding favors the spread of head lice.

Although transmission is not at all dependent on the socioeconomic status of individuals, the magnitude of lice infestation and the related household management are directly influenced by financial resources, access to treatment, and the availability of proper information. This might explain why head lice prevalence in poor rural communities and urban slums is between 28% and 43% in Brazil,^3,4^ and the prevalence at different schools in Norway ranged from 0% to 7.14%, although 36.43% of participating households had previously experienced a head lice infestation.^5^ The lack of health literacy and financial resources perpetuates the presence of head lice in families living in Brazilian slums, while in Norway it might represent a minor event during childhood.

For children aged 312 (preschool and elementary school), head lice infestation leads to high levels of anxiety and embarrassment, and low self-esteem.^2^ Itching is the most common symptom in individuals who are sensitive to the antigenic components present in louse saliva.^6^ Head lice bites tend to induce concentration problems, sleeplessness, head wounds, and scratches. Therefore, children from low-income families face health inequalities as they remain almost their whole childhood infested.

We launched a theater-based program intending to tackle the social stigma associated with head lice using scientific information and an affordable Brazilian solution based on a traditional medicine containing water, salt, and vinegar. This was a qualitative study that was designed to obtain meaningful information that would inform future interventions in this community.

## Methods

### Participants

The program, a community-based approach, was implemented at Maria Augusta Canto Camargo School in the city of Santa Brbara d'Oeste in the state of So Paulo, Brazil. The institution hosted a total of 362 preschool and elementary-level children. All children in the school and their families were eligible for the program.

### Instrumentation

A theater group prepared a comedy play, composed a satiric song, and set up an interactive festive show to dedramatize the finding of little head inhabitants and destigmatize those hosting them while providing information on head lice life cycles and the related public health issues. After this first encounter, parents joined the program and received a product to treat all the members of the family where pediculosis had been detected, in particular among children. Individuals with head lice were encouraged to fill in a satisfaction survey to help assess the program and the treatment protocol. At the same time, during the week, teachers in all classrooms addressed topics related to head lice with the children. The information was given and adjusted according to the different age groups. Teachers and students discussed scientific information on the life cycle of head lice, its management in the household, and the related social stigma.

### Procedure

**FIG. 1.**
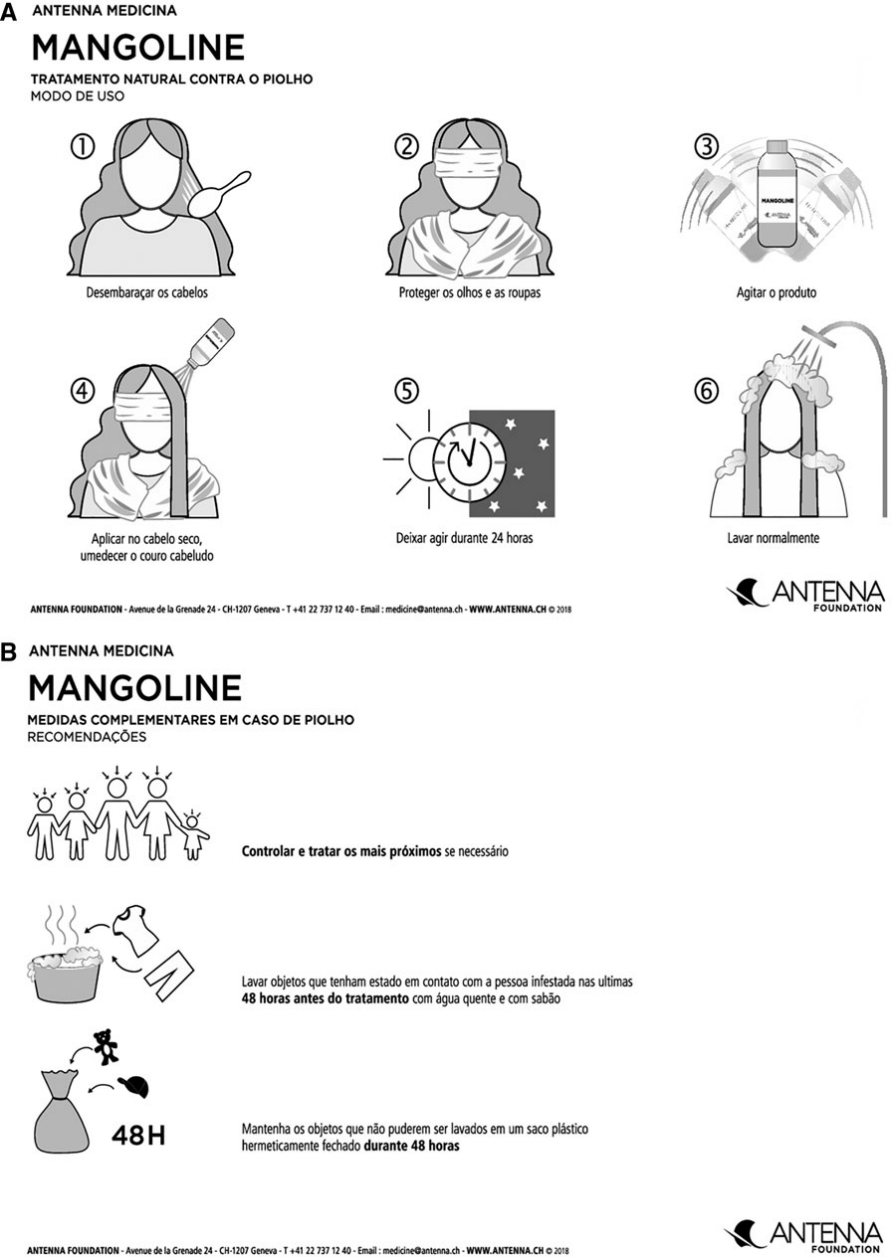
**(A)** Instructions on how to use the product: **(A.1)** untangle hair; **(A.2)** protect eyes and clothing; **(A.3)** stir the product; **(A.4)** apply to dry hair until moistened; **(A.5)** let it act for 24h; **(A.6)** wash normally. **(B)** Supplementary measures: control and treat those closest to you if necessary; wash objects that have been in contact with the infested person within 48h before treatment with hot water and soap; keep items that cannot be washed in a hermetically sealed plastic bag for 48h.

We encouraged school children identified as having pediculosis to undertake a treatment protocol: 4 applications of the lotion for 2 weeks. The first application took place at day 0; the second at day 2; the third at day 7 (1 week of treatment); the finalfourthtreatment took place at day 14 (2 weeks of treatment). The application method and procedure consisted of applying the product on dry hair such that it became wet. The treatment lasted 24h. The next day, the child could wash the hair as usual. No antilice combing was used during the test period of 14 days because it was first deemed nonfeasible by some partners of the program (although we had to change our mind afterward, this resulted in an estimate of the efficacy of the solution by itself). Parents and caregivers received additional information on how to control and treat infested household members, how to wash personal items that could have entered into contact with the affected individuals with hot water and soap, and how to confine nonwashable personal belongings to a plastic bag for 48h to eliminate head lice. All households received written instructions and a visual chart (Fig. 1A) explaining the different treatment steps and how to deal with personal belongings (Fig. 1B).

A solution based on a traditional Brazilian remedy to treat head lice was developed. The solution helped cover the prominent odor of vinegar by using a small amount of lavender essential oil to increase treatment adherence. Its final composition was as follows: 5g of sodium chloride, 10mL of vinegar (4% acetic acid), 0.5mL of baby shampoo, 2 drops of lavender oil, and enough water to make 100mL. This formulation was pretested with individuals in Switzerland (21 cases), Brazil (7 cases), and Ethiopia (12 cases). The pretests enabled us to adjust the formula and its number of applications based on user feedback.

The diagnosis for head lice was made by examining scalps, the direct visual inspection of preferred sites: neck and behind the ears. The examination was performed by trained school teachers and under the supervision of a parent or a caregiver. Children with eggs and/or adult lice and/or nymphs were considered to have pediculosis. No further investigation was conducted to differentiate viable from nonviable eggs.

Following the treatment, participants were asked to fill in a satisfaction survey. The survey aimed to assess the following: the presence of an adverse reaction (possible side effects of the product); compliance with the proposed application procedure; and the overall degree of satisfaction with the treatment. The whole community attended the program, and a total of 280 individuals participated in the study and returned the survey.

### Data analysis

Before treatment, all school children were observed to assess the presence of head lice. Individuals infested with adult head lice and/or eggs received enough treatment for themselves and their household members. Parents and caregivers received specific training to identify head lice and how to apply the solution. Questionnaires were also distributed to peripheral family members, should they wish to participate in the study. After treatment, its efficacy was assessed through a questionnaire distributed to all members of the household of the child presenting head lice infestation and trained school teachers confirmed the diagnosis. The study complied with the criteria for the Standards for Reporting Qualitative Research (SRQR).

## Results

The program included 280 participants (students, their parents, and siblings) belonging to a low-income socioeconomic group. Among them, 24% (67 participants) had head lice, with girls representing 85% of cases; 74.7% (*N*=50) of the participants infested with head lice were between the ages of 4 and 10; 7 participants were older than the age of 18, suggesting that other household members, such as parents, grandparents, and older siblings, also had head lice and used the product. Those individuals were included in the analysis, although they had not been examined by trained staff before and after treatment to keep them from being embarrassed. Among the participants with head lice nits and/or adults (67 out of 280), the number of participants with nits and adults dropped from 70.2% (*N*=47) to 19.4% (*N*=13), and 55.2% (*N*=37) of the participants who had pediculosis before treatment showed no signs of nits or adult lice after treatment. The number of participants showing only nits before treatment dropped from 19.4% to 12% after treatment. However, those showing only adult lice increased from 10.4% before treatment to 13.4% after treatment. Overall, among the participants infested with nits and/or adults (*N*=67), a rate of 55.2% (*N*=37) showed no signs of nits or adult lice after the program. Around 20% of total participants (*N*=13) applied the lotion only once instead of four times as initially prescribed. Among them, 11 stopped the treatment after the first application because they did not find any more head lice and 2 stopped it because they believed that it would not work. The immediate success of the proposed treatment or a participant's doubt about its efficacy was associated with several aspects, such as family structure and the time allocated to the study. For example, in families where the children rotate between each parent's home (cases of divorce and separation) or in families where both parents work, there was not enough time for them to get involved in the study. Hence, and concerning this group, only 25% of participants complied with the treatment up to completion (the four required applications of the lotion for 2 weeks). All participants from the school were checked after the 14th day, even those who did not complete the treatment; 72% of participants did not experience any side effects; some others reported mainly minor reactions, such as a burning sensation at the site of application of the lotion.

## Discussion

The whole-school approach is a socially inclusive method grouping and inviting all families in the school's neighborhood to discuss local health issues. The benefits of this method had previously been demonstrated in the management of head lice^7^ and the prevention of child obesity.^8^ Despite its potential in meeting children's basic health needs, this type of program is very rare in low-income settings.^9^

Our findings for the prevalence of head lice are in accordance with the literature regarding gender (among all those infested, 85% were girls) and age groups (74.7% were between 4 and 10 years old). Regardless of the socioeconomic status, girls account for the majority of affected individuals.^5,10^ This can be explained by the length of the hair and the fact that girls of this young age are found to have closer contact with each other than boys do.^11^ Although such factors influence the prevalence and the spread of head lice, the clustering of school children into classes is found to be more influential than individual characteristics such as age, gender, hair length, and even the size of the household. Previous studies also show that repeated infestations are more likely in dense populations, indicating that the density of hosts or groups of hosts influence transmission rates.^5^ Considering that families in low-resource communities share small households, this could be a major factor favoring infestation.^12^ This might explain the 24% overall head lice prevalence observed in our study as the children come from low-income families who share small households favoring the infestation.

The active involvement of school children, teachers, school workers, families, and the local community at large was a determinant in the successful implementation of the whole-school approach. Program adherence was higher than observed elsewhere,^5,7^ with 273 school children and 7 household members in a school that counted 362 students in total. The higher participation observed was probably because embarrassment related to social stigma, and the financial struggles in accessing safe and effective treatment were reported as the main barriers to treat head lice by low-income families.^13^ In our program, the community was empowered once it received sound scientific information about head lice management through a theater play and could afford a product to treat the family members. To overcome health inequality related to head lice infestation, those are very important elements when addressing low-income communities.

Acidic shampoos (pH 4.55.5) and formulations containing acetic acid and formic acid helped remove nits. Some authors claim that acidic solutions smooth out the surface of the hair, making nit and adult lice removal by metal comb easier, while others claim that the enhanced removal of the nits would be the result of loosening the hold the nits have on the hair.^1416^ However, this approach was also questioned and a nonclinical experiment showed that exposure to several formulations from the market that used a low pH was not efficient in removing nits.^17^ The action mechanism of a sodium chloride hypertonic solution is not fully known, but a rupture of the gastrointestinal tract was observed with its application. Desiccation is the suggested mode of action.^18,19^

A study of a whole-school approach conducted in the United Kingdom, Belgium, and Denmark shows that using a cost-effective treatment such as wet combingwhich consists of using a conditioner and the systematic use of a comb (with teeth <0.3mm apart) in a coordinated community program to eradicate head liceis efficient regardless of social inequalities.^7^ In a pilot trial, a 53% success rate at first use and total eradication in all families assigned the treatment were achieved by day 24 after using a wet-combing method.^20^ Although it is pesticide-free, wet combing is time-consuming and a high level of persistence is needed to eradicate head lice. Therefore, we used a similar approach to evaluate if a local traditional medicine would benefit a low-resource community. Our results show that 37 out of 67 participants were able to completely eradicate head lice using the solution without an antilice comb. The increased number of individuals presenting only adult head lice and no nits after having been treated (10.413.4%) may well reflect the new introduction of head lice from untreated individuals, reinforcing the need to coordinate this program with the whole community. Or it may indicate that the ovicidal effectiveness of the solution is higher than previously observed *in vitro*.^18^

Although this is still a modest success rate, 55.2% in comparison with formulations containing chemical pesticides such as benzyl alcohol, which had a 75% success rate in an open-label phase II trial,^21^ or a formula containing sodium chloride 1%, which saw an 85% success rate without combing,^19^ or even bug busting performed by nurses in a controlled situation,^7,20^ we point out that we observed no safety concerns in the population tested. In addition, the building of resistance to the product is unlikely in the future with this formulation and the use of an appropriate nit comb could come in handy as a complement to the product's application since it facilitates the removal of adult head lice. We will study this aspect in more depth as the program develops.

### Limitations

Our survey showed that 24% of the community had pediculosis. In the literature, a thorough visual inspection may only identify up to 50% of infestations,^22^ while the presence of nit shells alone indicates a recent history of infestation but does not confirm the active disease. In our study, we considered that children with nit and/or adult lice had pediculosis. This might represent a study limitation as some of the school children could have been treated even if they did not have pediculosis and some others might not have joined the program because head lice infestation could not be identified during the visual inspection. In addition, household members of children identified with pediculosis at school may have followed the treatment protocol but may not have felt comfortable filling out the survey because they were afraid of being identified and stigmatized. Therefore, a more precise and thorough detection method could have resulted in the measuring of a higher prevalence of head lice among the community, well above the 24% surveyed. Finally, the study aimed to be a pilot qualitative intervention, so cautious inferences should be made in terms of head lice prevalence among the population and also the efficacy of the head lice solution applied.

## Conclusions

With nearly 25% of individuals within the community affected, head lice infestations appear as a significant community health issue at the school where the study was conducted. This experience suggests that the use of playful activities associated with a well-known and accessible local product to treat head lice in low-income families gathered a high degree of community adherence and may be an important tool in overcoming health inequalities. Full measurement of the impact of the whole-school approach is necessary to better demonstrate the underlying potential and benefits of the program in the community.

## Human Subject Approval Statement

We obtained written informed consent from the parents or guardians of each child. The school director and the Secretary of Education of Santa Brbara d'Oeste granted permission to perform the intervention at the Maria Augusta Canto Camargo School.
